# Glucose 6-P Dehydrogenase—An Antioxidant Enzyme with Regulatory Functions in Skeletal Muscle during Exercise

**DOI:** 10.3390/cells11193041

**Published:** 2022-09-28

**Authors:** Esther García-Domínguez, Aitor Carretero, Aurora Viña-Almunia, Julio Domenech-Fernandez, Gloria Olaso-Gonzalez, Jose Viña, Mari Carmen Gomez-Cabrera

**Affiliations:** 1 Freshage Research Group, Department of Physiology, Faculty of Medicine, University of Valencia and CIBERFES, Fundación Investigación Hospital Clínico Universitario/INCLIVA, 46010 Valencia, Spain; 2 Centro de Salud San Isidro, Consorcio Hospital General Universitario de Valencia, 46014 Valencia, Spain; 3 Health Care Department Arnau-Lliria, Servicio de Cirugía Ortopédica y Traumatología, Hospital Arnau de Vilanova y Hospital de Liria, 46015 Valencia, Spain

**Keywords:** G6PD, pentose phosphate pathway, NADPH, skeletal muscle, physical training, aging

## Abstract

Hypomorphic Glucose 6-P dehydrogenase (G6PD) alleles, which cause G6PD deficiency, affect around one in twenty people worldwide. The high incidence of G6PD deficiency may reflect an evolutionary adaptation to the widespread prevalence of malaria, as G6PD-deficient red blood cells (RBCs) are hostile to the malaria parasites that infect humans. Although medical interest in this enzyme deficiency has been mainly focused on RBCs, more recent evidence suggests that there are broader implications for G6PD deficiency in health, including in skeletal muscle diseases. G6PD catalyzes the rate-limiting step in the pentose phosphate pathway (PPP), which provides the precursors of nucleotide synthesis for DNA replication as well as reduced nicotinamide adenine dinucleotide phosphate (NADPH). NADPH is involved in the detoxification of cellular reactive oxygen species (ROS) and de novo lipid synthesis. An association between increased PPP activity and the stimulation of cell growth has been reported in different tissues including the skeletal muscle, liver, and kidney. PPP activity is increased in skeletal muscle during embryogenesis, denervation, ischemia, mechanical overload, the injection of myonecrotic agents, and physical exercise. In fact, the highest relative increase in the activity of skeletal muscle enzymes after one bout of exhaustive exercise is that of G6PD, suggesting that the activation of the PPP occurs in skeletal muscle to provide substrates for muscle repair. The age-associated loss in muscle mass and strength leads to a decrease in G6PD activity and protein content in skeletal muscle. G6PD overexpression in *Drosophila Melanogaster* and mice protects against metabolic stress, oxidative damage, and age-associated functional decline, and results in an extended median lifespan. This review discusses whether the well-known positive effects of exercise training in skeletal muscle are mediated through an increase in G6PD.

## 1. The Pentose Phosphate Pathway and the Regulation of G6PD

Glucose is catabolized by two pathways: glycolysis, to generate ATP, and the pentose phosphate pathway (PPP), also known as the hexose monophosphate shunt, to generate reduced nicotinamide adenine dinucleotide phosphate (NADPH) and ribose 5-phosphate (R5P) for nucleotide synthesis.

The PPP was fully elucidated in the 1950s owing to the joint work of several researchers including Efraim Racker [1], Bernard Horecker [2], and Frank Dickens [3]. However, in the 1930s, Otto Warburg was the first to provide evidence of the existence of the PPP when he studied the oxidation of glucose 6-phosphate (G6P) to 6-phosphogluconate (6PG) and discovered NADP^+^ [4].

**Figure 1:**
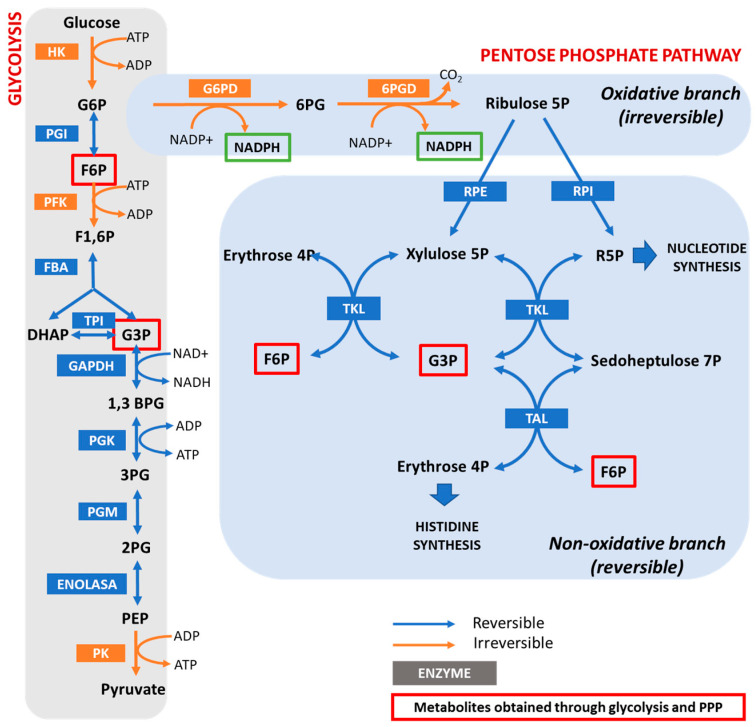
The pentose phosphate pathway and its interrelation with glycolysis. Blue and orange arrows show reversible and irreversible reactions, respectively. Enzyme names are shown in blue and orange boxes. Glycolysis metabolites obtained through the PPP are shown in red squares. G6P—glucose 6-phosphate; F6P—fructose 6-phosphate; F1,6P—fructose 1,6-biphosphate; DHAP—dihydroxyacetone phosphate; G3P—glyceraldehyde 3-phosphate; 1,3 BPG—1,3-bisphosphoglycerate; 3PG—3-phosphoglycerate; 2PG—2-phosphoglycerate; PEP—phosphoenolpyruvate; 6PG—6-phosphogluconate; HK—hexokinase; PGI—phosphoglucoisomerase; PFK—phosphofructokinase; FBA—fructose-1,6-bisphosphate aldolase; TPI—triose-phosphate isomerase; GAPDH—glyceraldehyde 3-phosphate dehydrogenase; PGK—phosphoglycerate kinase; PGM—phosphoglycerate mutase; G6PD—glucose 6-phosphate dehydrogenase; 6PGD—6-phosphogluconate dehydrogenase; RPE—ribulose-phosphate 3-epimerase; RPI—ribose-5-phosphate isomerase; TKL—transketolase; TAL: transaldolase.

The PPP occurs entirely in the cytosol and has both anabolic and catabolic functions. It takes place through two phases: the oxidative branch, which is irreversible, and the non-oxidative branch, which is reversible [5] (See Figure 1).

The PPP competes with glycolysis for the catabolism of glucose 6-P (G6P). In the oxidative PPP branch, G6P is converted to ribulose-5P with the loss of CO_2_ and the formation of two NADPH molecules. Glucose 6-P dehydrogenase (G6PD) catalyzes the first committed step of this PPP branch, which involves the conversion of G6P to 6PG and the generation of the first NADPH molecule. This irreversible reaction is unique to the PPP and has a primary role in the regulation of this pathway [6]. NADPH can be also synthesized by other enzymes such as the NADPH-malic enzyme, NADPH-dependent isocitrate dehydrogenase, and transhydrogenases [7].

**Figure 2:**
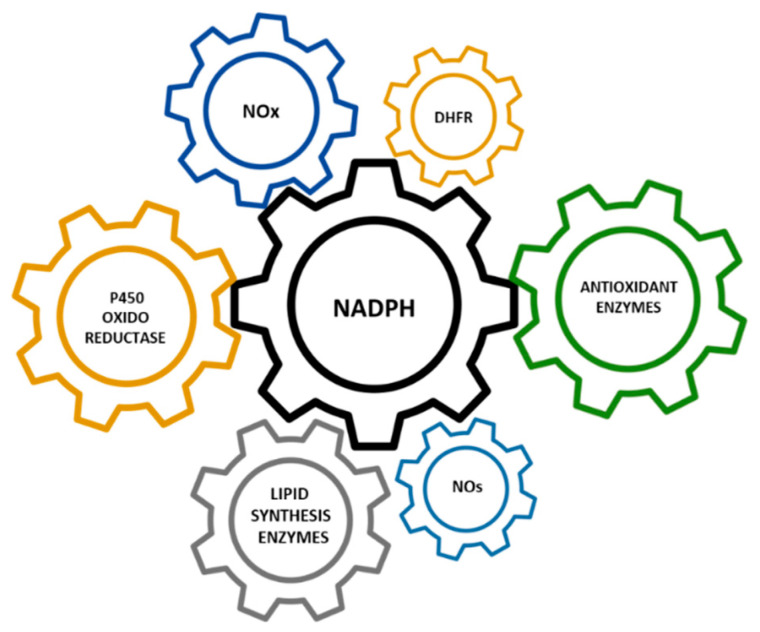
NADPH-dependent enzymes. NOs—nitric oxide synthases; NOx—NADPH-oxidase enzymes; DHFR—dihydrofolate reductase.

Across all forms of life, NADPH donates high-energy electrons for reductive biosynthesis and antioxidant defense [8]. One of the main functions of NADPH in our cells is in the maintenance of redox homeostasis [9]. NADPH is the electron donor for the antioxidant enzymes glutathione reductase (GR) and thioredoxin reductases (TrxR). Reduced glutathione (GSH) and reduced thioredoxin (Trx(SH)_2_) provide reducing equivalents for glutathione peroxidase (GPx), glutaredoxins (Grx), and peroxiredoxins (Prx). Thus, NADPH is located at the core of the antioxidant defense [10]. Another function of the pyridine nucleotide NADPH is to boost biosynthetic reactions in our cells. It provides the reducing power for fatty acids and cholesterol synthesis [9]. Finally, NADPH acts as the coenzyme of NADPH-oxidase enzymes (NOXs), which—through the generation of the superoxide radical—are involved in the oxidative burst and its defensive functions in our immune cells (granulocytes and macrophages) [11], and even in other cellular types [12,13]. Nitric oxide synthases (NOS), dihydrofolate reductase (DHFR), and cytochrome P450 oxidoreductase are also NADPH-dependent enzymes (Figure 2).

**Figure 3:**
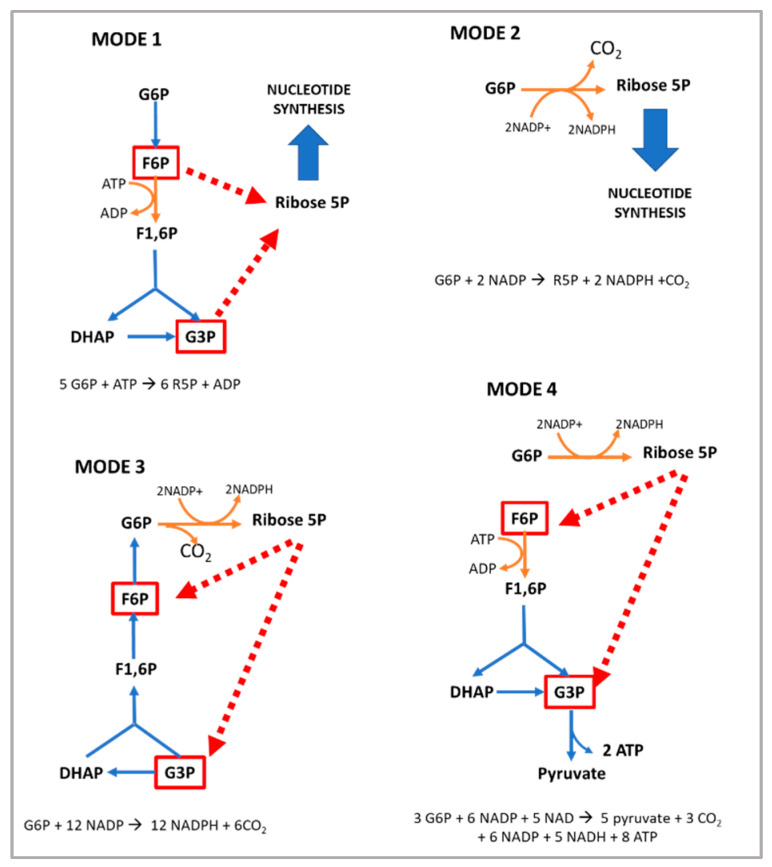
Pentose phosphate pathway regulation depends on cellular needs. **MODE 1:** This mode dominates when the need for R5P is higher than that for NADPH, for instance, in proliferative cells. In this situation, the glycolytic metabolites 3GP and F6P can be converted in R5P through the reversible non-oxidative PPP. The oxidative PPP and its associated NADPH formation are bypassed. **MODE 2**: This mode occurs when the needs for NADPH and R5P are balanced. Then, ideally, from one molecule of G6P two molecules of NADPH and a molecule of R5P can be obtained with no generation of glycolytic metabolite. **MODE 3**: This mode is adopted when the cellular need for NADPH exceeds that for R5P and ATP, for instance, during fatty acid synthesis in adipocytes. The non-oxidative phase of the pathway leads to the conversion of ribulose 5-phosphate to fructose 6-phosphate (F6P) and glyceraldehyde 3-phosphate (G3P). Then, these glycolytic metabolites—through gluconeogenesis reactions—form G6P, which can enter again into the PPP to produce more NADPH. **MODE 4:** In this scenario, the cellular need for NADPH and ATP is higher than that for R5P. As described in PPP mode 3, ribulose 5-P is transformed into G3P and F5P through the non-oxidative branch of the PPP; however, in mode 4, these molecules are metabolized to pyruvate through glycolysis, which is associated with ATP formation.

The non-oxidative PPP is a flexible pathway that is able to adapt to varying cellular needs through the generation of different phosphorylated carbohydrates with three, four, five, or seven carbons [14]. This branch begins with a bifurcation: the ribulose-5P obtained from the oxidative PPP after epimerization is transformed into xylulose 5-phosphate or can isomerize and form ribose-5-phosphate, which can be used for nucleotide synthesis. The main modes of the PPP depending on cellular needs are summarized in Figure 3 [15]. Although the corresponding stoichiometric reaction for each mode is shown, the carbon flux is difficult to quantify in cells [15], and a situation in which all the flux is directed to one is unlikely. On the other hand, mode 3, also known as the recycling PPP mode, uses steps from gluconeogenesis; therefore, it only can take place in cells containing fructose biphosphatase. Mode 4 represents the standard operation of the pathway.

As previously mentioned, G6PD is the key enzyme in the regulation of the PPP. Therefore, any factor able to modify the level or activity of G6PD will determine the flow of the PPP. The “coarse control” of the PPP is carried out by modifications in the levels, location, and activity of G6PD [16]. Factors such as diet composition can induce changes in the synthesis of G6PD [17,18]. An excess of carbohydrates in the diet leads to lipogenesis and the deposition of fat, which is associated with a 5–10-fold increase in G6PD activity in the liver [16]. Accordingly, the expression of the G6PD gene is upregulated by the major transcription factor sterol-responsive element binding protein [19]. High insulin and low glucagon levels, which are associated with this kind of diet, have also been described as regulators of G6PD through the control of mRNA synthesis [20]. The transcription factor Nrf2, which regulates the antioxidant cellular response, also enhances G6PD gene expression [21]. Interestingly, diets rich in polyunsaturated fatty acids (PUFAs) have the opposite effect on G6PD levels.

However, the main regulating factor for G6PD activity is the NADPH/NADP^+^ ratio. NADPH is a competitive inhibitor of G6PD [22], while NADP^+^ is required to maintain the conformation of G6PD and thus its catalytic activity [23]. If the NADPH/NADP^+^ ratio is increased, G6PD activity is lowered, and vice versa. In vitro experiments have shown no G6PD activity with NADPH/NADP^+^ ratios close to 10 [16]. The fact that the NADPH/NADP^+^ ratio in physiological conditions is high (70–300 depending on the tissue) [24] led Egglestone and Krebs to postulate the existence of a mechanism able to modulate the inhibition of G6PD by NADPH [16]. These authors, after testing the effect of over a hundred cell constituents, demonstrated that the physiological concentrations of oxidized glutathione (GSSG) and AMP were able to counteract the inhibition of G6PD by NADPH [16]. The inhibitory effect of GSSG on NADPH could not be attributed to GR activity since a complete inhibition of the enzyme did not abolish the GSSG effect. Recently, a similar role of GSSG in the “fine control” of the PPP has been assigned to a 100 kDa protein named CRING (cofactor that reverses the NADPH inhibition of G6PD). GSSG is required for CRING function. CRING is found in specific tissues including adipose, liver, and adrenal tissues [7]. Finally, the post-translational modification of G6PD also plays a role in its activity, as is the case with G6PD phosphorylation by the nonreceptor tyrosine kinase Src. As a consequence, the inhibition of Src causes reduced G6PD activity in endothelial cells [25].

Apart from the NADPH/NADP^+^ ratio, several other factors have been described as positive or negative regulators of G6PD; they are included in the last section of this manuscript.

## 2. Loss of Function Models for G6PD

G6PD deficiency is the most common human enzymopathy. It is very heterogeneous and was first described in humans by Marks and Gross in 1959 [26]. Approximately 400 million people worldwide carry a mutation in the G6PD gene, which causes an enzyme deficiency. Deficient alleles are prevalent in South and North America and in northern Europe [27]. However, the highest prevalence of this enzymopathy is reported in Africa, the Middle East, the central and southern Pacific Islands, southern Europe, and southeast Asia. The global distribution of the G6PD deficiency is strikingly similar to that of malaria. In areas where G6PD deficiency is common, *Plasmodium Falciparum* malaria is endemic, supporting the so-called malaria protection hypothesis [28]. Epidemiological evidence for the association between G6PD deficiency and a reduction in the risk of severe malaria [29] has been accompanied by the results of in vitro work showing that parasite growth is slowest in G6PD-deficient cells [28].

It has also been shown that G6PD-deficient red blood cells (RBCs) infected with parasites undergo macrophage-induced phagocytosis at an earlier stage of *Plasmodium Falciparum* maturation than normal RBCs. This could be a further protective mechanism against malaria [30]. The vulnerability of RBCs to mutant G6PD may reflect their lack of mitochondria and thus their inability to endogenously produce the substrates for malic enzyme and isocitrate dehydrogenase [8]. This may also reflect RBCs lack of nuclei and failure to replace the deficient G6PD protein as the cells age.

The G6PD gene is located at the telomeric region in the X chromosome. Thus, its deficiency is an X-linked hereditary defect that causes variants with different clinical phenotypes (about 140 mutations have been described). The G6PD-encoding gene has been well preserved throughout evolution [31]. As a monomer, the protein is inactive; however, as a dimer or tetramer, it is active. In its catalytic center, there is an amino acid sequence that binds to NADPH. The deficiency is caused by protein instability due to amino acid substitutions in different enzyme locations [28]. The diagnosis of G6PD deficiency is based on the spectrophotometric quantification of the enzyme’s activity [32]. There are five categories of G6PD deficiency based on clinical manifestations and enzyme activity (Table 1) [28].

The most frequent clinical manifestations of G6PD deficiency are acute and chronic hemolytic anemia and neonatal jaundice [28]. The prevention of hemolysis by avoiding oxidative stress represents the most effective management of G6PD deficiency. Oxidative stress can be triggered by agents such as drugs (primaquine, sulfonamide, or acetanilide), infections (hepatitis viruses, cytomegalovirus, or pneumonia), or the ingestion of fava beans (favism). Favism is a hemolytic response to the consumption of fava beans that takes place in some individuals with G6PD deficiency [33]. Isouramil, divicine, and convicine are thought to be the toxic constituents of fava beans that lead to the onset of the clinical manifestations of deficiency [28]. The mechanism by which increased sensitivity to oxidative damage leads to hemolysis has not been fully elucidated [34].

Several clinical disorders, such as diabetes and myocardial infarction, precipitate hemolysis in G6PD-deficient subjects [35,36].

G6PD is ubiquitously expressed in mammalian cells, with the highest expression observed in the immune cells, testes, adrenals, and brain [37]. It is often upregulated in tumors [9,38]. The enzyme is subject to tissue-specific transcriptional regulation, which in turn is correlated with the methylation of specific sites in the gene [37]. We recently found that immune cells, and especially T cells, are dependent on G6PD to maintain NADPH levels and effector functions [8]. Activated T cells do not express substantial levels of malic enzyme or isocitrate dehydrogenase and produce NADPH mainly through the PPP, which is sharply upregulated during T cell activation and is related to pro-inflammatory cytokine production [8]. Thus, severe G6PD mutations that affect the enzyme’s catalytic ability can present as immune deficiency [39].

Favism has a higher incidence in males than females [28]. Males are hemizygous for the G6PD gene and thus can have normal gene expression or be G6PD deficient. Females, with two copies of the G6PD gene on each X chromosome, can have normal gene expression, be homozygous, or be heterozygous. Heterozygous females can achieve the same degree of G6PD deficiency and can be susceptible to the same pathophysiological phenotype present in G6PD-deficient males. However, heterozygous women on average have less severe clinical manifestations than G6PD-deficient males [28].

The World Health Organization Scientific Group has emphasized the need to develop animal research models for this frequent human hereditary disorder. Genetically, G6PD knockout mice are not viable [40]. Mouse viability is dependent on G6PD activity, as evidenced by a decrease in litter size corresponding to a decrease in G6PD activity [41].

In 1988, Merkle and coworkers created the first X-linked G6PD deficient animal model using 1-ethyl-l-nitrosourea-induced chemical mutagenesis [42]. Williams’ research team reported a single point mutation (A to T transversion) at the 3′ end of exon 1 that explained the decrease in G6PD activity in the G6PD-deficient mice [43].

Heterozygous, hemizygous, and homozygous mutants have ~60%, ~15%, and ~15% of remaining precipitate activity in RBCs, respectively, when compared to wild type (WT) mice. Therefore, in comparison with the human classification of G6PD mutations, the mouse mutant falls into class III (mild mutation severity) with respect to its hematological and biochemical characteristics.

Using this model, it has been shown that mild G6PD deficiency (15% activity of WT) induces a pronounced decrease in RBC deformability and worsens erythrocyte dysfunction during sepsis. RBC dysfunction aggravates organ dysfunction and microcirculatory disturbances and may also contribute to the modulation of macrophage responses during severe infections in G6PD-deficient animals [43,44].

A significant number of studies have unveiled the roles of G6PD in various aspects of physiology other than erythrocytic pathophysiology, such as diabetes, cardiovascular disease, and neurodegeneration [45]. The association between G6PD deficiency and the development of diabetes has been supported by epidemiological studies conducted in different research groups and populations [46,47,48]. An increased risk for diabetes, and also of diabetic complications such as proliferative retinopathy [49], has been reported in G6PD-deficient subjects [50,51].

In preclinical studies, it has been shown that the liver and pancreas of diabetic rats show a reduction in G6PD activity [52]. Pancreatic islets from G6PD mutant mice are smaller than those of WT mice [53], which suggests that G6PD plays important roles in the survival and functions of pancreatic cells. Accordingly, it has been reported that mutations in the G6PD gene and the consequent drop in G6PD activity are sufficient to cause changes similar to those seen in diabetic mice [54]. Using the opposite methodological approach, we found that G6PD transgenic (Tg) mice moderately overexpressing the enzyme (2–4-fold overexpression) were more insulin sensitive and glucose tolerant than WT controls [9]. These results are in accordance with previous mouse overexpression models of NADPH-dependent ROS-detoxifying enzymes. For instance, Prx3-Tg and Prx4-Tg mice were shown to have better insulin sensitivity and glucose tolerance compared to WT mice [55]. Although the molecular mechanism underlying the association between G6PD deficiency and diabetes is not completely understood, current evidence suggests that G6PD deficiency may be a risk factor for diabetes, with higher odds among men compared to women [46,47].

The role of ROS as physiological signals as well as pathological stresses has been demonstrated repeatedly in the cardiovascular system [56,57,58,59]. However, the relation between G6PD deficiency and risk for cardiovascular disease and subsequent outcomes is unclear. The existing data indicate a complex interplay in which the adverse effects of G6PD deficiency may outweigh the potential protective effects in the context of cardiac stress [34,60,61,62].

The risk of redox-mediated damage to brain cells in G6PD deficiency has also been studied [63]. G6PD is an important enzyme in the protection against age-associated ROS neurodegenerative effects, and more specifically in the age-associated increase in oxidative DNA damage in the brain [63]. Recently, brain damage associated with ROS production in G6PD-deficient animals was also found to have functional consequences. Old G6PD-deficient male mice exhibited synaptic dysfunction in their hippocampal slices while young and old G6PD-deficient females exhibited deficits in executive functions and social dominance [64].

Taken together, these results suggest that there are broad health implications of G6PD deficiencies. Among the potential outcomes related to G6PD loss of function, birth defects, heart disease, diabetes, and neurodegeneration are highlighted.

## 3. G6PD and Cell Growth

The modulation of cell survival and cell growth relies on intracellular redox regulation [65]. As mentioned in the previous sections of this manuscript, NADPH—the principal intracellular reductant—is a critical modulator of redox potential. In 1999, Dr. Stanton and coworkers found that G6PD plays an important role in cell death by regulating intracellular redox levels [66]. The inhibition of G6PD by both dehydroepiandrosterone (DHEA) and 6-aminonicotinamide (6-ANAD) augmented cell death triggered by serum deprivation and oxidative stress, while the overexpression of G6PD in a cell line conferred resistance to H_2_O_2_-induced cell death. Previously, in G6PD-deficient cell lines, it was reported that these cells had decreased cloning efficiencies and growth rates and were highly sensitive to ROS when compared to cells expressing endogenous levels of the enzyme [67]. Consistent with these results, an association between the stimulation of cell growth in different tissues and increased PPP activity has also been reported [68]. Kidney hypertrophy due to unilateral nephrectomy is associated with increased G6PD activity [69], while the growth of rat liver cells stimulated by growth hormone is also associated with an increase in G6PD activity [70].

In experiments to determine if the increased G6PD activity per se is an essential component of normal cell growth, it was found that G6PD activity was directly correlated with cell growth, that the inhibition of G6PD activity prevented growth, and that the overexpression of G6PD alone stimulated [3H]-thymidine incorporation [65].

As previously mentioned, cancers and cultured tumor cells exhibit large increases in G6PD activity [71]. To test the potential tumorigenic risk of G6PD overexpression, we crossed G6PD-Tg mice with several genetically modified tumor-prone animals, including ATM-KO (that develop T-cell lymphomas), Eμ-myc (that develop B-cell lymphomas), p53-KO (that develop T-cell lymphomas and sarcomas), and MMTV-PyMT (that develop mammary tumors) [9,10]. In all of these combinations, mice carrying a G6PD-Tg allele showed the same tumor latency and incidence as the WT mice. These data indicate that a moderate and regulated increase in NADPH levels or G6PD expression and activity does not result in increased tumor incidence [9]. On the contrary, G6PD-Tg mice showed improved lifespan and health parameters as they grew old: (i) the transgenic animals were more insulin sensitive and glucose tolerant; (ii) old G6PD-Tg mice tended to gain less weight and exhibited improved motor coordination; and (iii) G6PD-Tg females showed a ~14% increase in medium lifespan. At the molecular level, we found a reduction in age-associated lipid peroxidation and DNA oxidation in different tissues [9]. We related the decrease in age-associated oxidative damage to macromolecules, a result of the modulation of cellular NADPH levels, to the improvements in health and lifespan in the G6PD-Tg animals.

The treatment of both animals and humans with antioxidant vitamins and other supplements [9], specially at high doses, has not been shown to increase lifespan and has failed to protect against age-induced pathologies. Studies on the biological roles of ROS have uncovered the beneficial signaling functions of these highly reactive molecules to explain these contradictory results [10]. The overexpression of antioxidant enzymes vs. the administration of exogenous antioxidants are very different approaches to test the importance of redox balance both in aging and age-associated diseases with very different outcomes [10,55].

## 4. G6PD in the Regeneration of Skeletal Muscle after Damage

The hexose monophosphate shunt is considered an almost negligible pathway in normal muscle. For this reason, the function of G6PD in skeletal muscle has been poorly investigated.

In vitro studies have shown that, under normal conditions, glucose breakdown takes place via both the Embden–Meyerhof pathway and the PPP in the liver, pancreas, arterial wall, kidney, spleen, and adrenals. However, in the central nervous system and cardiac and striated muscle, it is metabolized mainly via the glycolytic route [72]. In addition, several conditions increase the activity of the PPP in skeletal muscle: (i) embryogenesis [73]; (ii) denervation; (iii) ischemia; (iv) hypertrophy; (v) the injection of myonecrotic agents with local degeneration effects [74,75]; and (vi) physical exercise [32].

The injection of myonecrotic agents (bupivacaine, Marcaine, or cardiotoxin) induces a rapid (8 h) and dramatic (6–9-fold) increase in the activities of G6PD and 6PGD during regeneration after muscle destruction. By using histological techniques [76,77], it has been shown that G6PD is localized within muscle cells in regenerating muscle; thus, the enhanced enzyme activity resides in the muscle fibers themselves for at least the first 6–8 h after Marcaine injection. After that time, phagocytic cells contribute to the increase in enzyme activity [74]. The enhanced activities of G6PD and 6PGD likely reflect accelerated glucose utilization for the production of nucleic acids and lipids [75,78,79,80]. In this regard, increased quantities of RNA have been noted in a number of studies on muscle regeneration [81,82,83]. The enhancement of the PPP is important for anabolic processes in the initial stages of skeletal muscle regeneration; however, the role of G6PD in skeletal muscle goes beyond biosynthetic processes. In 2016, Febbraio and coworkers found that one mechanism linking an altered cellular redox state to insulin resistance is NOS [84]. S G6PD activity in skeletal muscle is linked to nitric oxide (NO) bioavailability; thus, an impairment in the NOS isozyme (nNOSμ) in insulin resistant states in rodents and humans leads to an increase in G6PD activity [84].

The consequences of G6PD deficiency in skeletal muscle have been studied in clinical cases of rhabdomyolysis [85] and myopathies [86]. In fact, a statistically significant relationship has been found with regard to the activity of G6PD between RBCs and muscle in humans [87].

## 5. Positive Regulators of G6PD Activity in Skeletal Muscle—Role of Exercise

As previously mentioned, G6PD overexpression in *Drosophila Melanogaster* and mice protects against metabolic stress [9,88] and oxidative damage [9]. Very recently, we found that it also delays the onset of frailty by protecting against muscle damage [32].

As shown in Table 2, G6PD can be regulated by pharmacological, nutritional, and physiological interventions, such as physical exercise [68].

G6PD activity has been studied in both skeletal muscle and erythrocytes after one bout of exhaustive exercise. Surprisingly, contradictory results were found in the literature.

G6PD activity in erythrocytes is reduced in humans after one bout of high intensity exercise (~40%), likely due to ROS generation [89]. Accordingly, supplementation with L-cysteine for a week (0.5 g/24 h) [89] or with α-Tocopherol for a month (200 mg/24 h) [90] leads to the maintenance of the enzyme activity. These results have also been verified in long distance runners [91] and soccer players [91,92]. To the contrary, the highest relative increase in enzyme activities, both for mitochondrial and extramitochondrial enzymes, after exhaustive swimming in rat skeletal muscle was shown for G6PD and 6PGD, which increased by 115% and 40%, respectively, 1 and 3 days after an acute bout of exercise [93]. Similarly, an increase in muscle G6PD activity of ~100–350% was observed after a downhill running protocol in rats [94], suggesting that the activation of the PPP occurs in skeletal muscle to provide substrates for muscle repair.

In one study, the changes in G6PD expression in skeletal muscle associated with different exercise intensities were investigated [95]. Based on the lactate threshold, it was shown that low-intensity aerobic treadmill running induced higher increases in the mRNA levels of G6PD in rat soleus muscle when compared to high-intensity anaerobic running [95]. Exercise duration is also a critical factor in the activation of G6PD in skeletal muscle. A significant linear correlation has been reported between the duration of downhill running (0, 30, or 90 min) and G6PD activity in different muscle groups in untrained rats [94].

G6PD activity also shows a susceptibility to exercise training in skeletal muscle. The exercise-induced elevation in muscle G6PD activity after one bout of downhill running was shown to be significantly reduced with only 5 days of either level or downhill training in rats [94]. This is the reason why changes in skeletal muscle G6PD activity have been widely used to study the “repeated bout effect”, which refers to an adaptation whereby a single bout of eccentric exercise protects against muscle damage from subsequent eccentric bouts [94,96].

The activity of G6PD and 6PDG increases pronouncedly by a factor of three in the gastrocnemius muscle after 5 days of repeated ischemia [97]. A similar increase in the activities of PPP enzymes has also been found in the heart after myocardial infarction [98]. Again, these results suggest that the increase in G6PD activity is important for repair purposes, as it increases the production of NADPH and the pentoses necessary for biosynthetic processes.

The PPP has been proven to be a fundamental metabolic pathway that allows for rapid and robust hypertrophic growth in muscle cells in response to mechanical overload [99]. For example, the denervation of one half of the diaphragm was shown to induce transient hypertrophy in the muscle on the other side [100]. In this model, the activities of G6PD and 6PDG increased immediately after denervation, reaching a maximum after 3 days [100]. More recently, the importance of G6PD in the regulation of skeletal muscle metabolism during hypertrophy was highlighted in a study analyzing gene expression from a transcriptomic microarray of specific metabolic pathways in mechanically overloaded plantaris muscle-induced hypertrophy [99]. A robust increase in G6PD mRNA expression was found in the overloaded muscle throughout the whole analyzed time course (1, 3, 5, and 7 days), consistent with an increase in NADPH levels to support nucleotide biosynthesis and to boost the muscle antioxidant defense [99]. It was also shown that the abundance of the G6PD protein significantly increased (~140%) in response to 5 days of mechanical overload in muscle [101].

The “MyoMouse” is a conditional mice model that inducibly expresses an activated form of Akt1 specifically in skeletal muscle [102]. The induction of the Akt1 signaling pathway leads to selective hypertrophy in type II fibers and an increase in muscle strength [102,103]. A combination of metabolomic and transcriptomic analyses has shown that Akt1-induced muscle growth is accompanied by a robust upregulation of biosynthetic metabolic pathways, such as the PPP, and the downregulation of catabolic pathways, such as glycolysis and oxidative phosphorylation [103]. Specifically, the “MyoMouse” shows a 3.5-fold increase in G6PD and a 2.3-fold increase in 6PDG in the hypertrophied muscles. Consistent with an increase in metabolite flux through the PPP, a 1.8-fold accumulation of R5P, an increase in total RNA, and an increase in purines and pyrimidine metabolites, including 5-aminoimidazole-4-carboxamide ribonucleotide (AICAR) and xanthosine, were also reported in the muscle tissue [103].

A potential limitation of the studies discussed above is the fact that all the analyses encompassed the whole muscle and could not distinguish the contribution of non-muscle cell types to the observed changes in G6PD expression, protein levels, or activity.

It has been suggested that the accumulation of cells in the connective tissue rather than changes in the activity within muscle fibers may explain the increase in the activity of the PPP enzymes in skeletal muscle following injury [104]. Macrophage, neutrophil, and mast cell levels are elevated after exercise and in mechanically overloaded muscles [104,105], which may influence the reported activity of G6PD.

The development of in vitro studies in C2C12 myoblasts has helped to overcome this concern. The overexpression of G6PD in C2C12 G6PD cells promotes their proliferation and significantly increases the percentage of EdU-positive cells. To the contrary, G6PD inhibition in myoblasts induces cell cycle arrest in the G0/G1 phase and suppresses muscle cell proliferation [106]. Moreover, the high-frequency electrical stimulation of C2C12 myotubes—mimicking muscle contraction—increases the expression of genes encoding the enzymes of the PPP [107].

The results reported by McCarthy’s research team also provide evidence that most of the observed changes in gene expression reflected in skeletal muscle occur within muscle fibers themselves [108]. These authors showed that myofibers are overwhelmingly the most transcriptionally active cell type in skeletal muscle at rest and during muscle hypertrophy. Approximately 90% of nascent RNA is associated with myonuclei during a mechanical overload induced by synergist ablation.

The results reported by Shimokawa and coworkers also support the idea that the exercise-induced increase G6PD activity is muscle specific and independent of inflammatory cells [95]. They found an increase in the mRNA expression of G6PD with aerobic exercise in rat soleus muscle, while this increment was absent in the animals following an anaerobic protocol. Macrophage invasion and injured and regenerating fibers were observed after anaerobic exercise, while neither of these signs of damage were found after the aerobic protocol [95]

Finally, testosterone [109] and growth hormone-induced muscle fiber hypertrophy in aging [110], two treatments that are independent of inflammatory signals, have been associated with an increase in G6PD protein levels in skeletal muscle.

These data suggest that muscle adaptations to exercise training or to mechanical overload require enhanced redox metabolism via the production of NADPH through the PPP and an increase in the expression of G6PD [99,111].

Physical exercise acutely increases ROS generation; however, if practiced regularly, it induces positive adaptations in mitochondrial density [112,113,114,115] and antioxidant defenses, including increased G6PD enzymatic activity [115,116,117]. Growing evidence suggests that physical training upregulates the level of antioxidant enzymes in the tissues actively involved in exercise [118,119]. For instance, eccentric exercise training in mice lasting 5 days was shown to be enough to induce an increase in G6PD mRNA levels and activity in skeletal muscle in young animals [32], similar to that found in a transgenic mouse model moderately overexpressing G6PD [9].

The age-associated loss in muscle mass and strength (i.e., sarcopenia) leads to a decrease in G6PD activity and protein content in skeletal muscle [120]. Whether the well-known positive effects of exercise training in old individuals are mediated through an increase in G6PD activity should be further studied in depth. The results published to date are contradictory and do not allow definitive conclusions to be drawn [121,122,123,124].

Finally, the question of whether exercise training is a safe and useful intervention in G6PD-deficient patients is something that has been an object of debate. G6PD-deficient individuals, as previously mentioned, are less protected against oxidative stress and could be predisposed to oxidative damage when they perform high-intensity physical training [125]. However, several studies have shown that exercise intensity does not cause oxidative stress or hemolysis above those levels expected in people without G6PD deficiency [126,127,128,129]. Therefore, despite the limited published studies, it seems that G6PD-deficient patients can safely participate in physical exercise programs with different intensities and durations.

**Table 2:** Cellular signals regulating G6PD and the PPP.

Positive Regulators	Negative Regulators
Acetylation [130]	5′ adenosine monophosphate-activated protein kinase (AMPK) [131]
G6PD activator AG1 [132]	Aldosterone [133]
AKT [134]	Angiotensin II [120]
ATM serine/threonine kinase (ATM) [135]	Arachidonic acid [136]
Benfotiamine (vitamin B1 analog) [110,137]	Cyclic adenosine monophosphate (cAMP) [138]
Proto-oncogene tyrosine-protein kinase Src (c-Src) [25]	cAMP-dependent protein kinase A [138]
cGMP-dependent protein kinase G [139]	cAMP response element modulator (CREM) [133]
Cyclin D3-CDK6 [140]	Dehydroepiandrosterone (DHEA) [141]
Epidermal growth factor (EGF) [142]	miR-122 and miR-1 [143]
Estrogens [110]	p38 mitogen-activated protein kinase [136]
Exercise [32]	p53 [144]
Glycosylation [145]	Phosphatase and tensin homolog (PTEN) [146]
Growth hormone [110]	TP53 [144]
Hepatocyte growth factor (HGF) [147]	Tumor necrosis factor-α (TNFα) [68]
Heat shock protein 27 (Hsp27) [148]	
Hypoxia inducible factor (HIF) [149]	
Inhibitor of DNA binding 1 (ID1) [150]	
Insulin [151]	
Mammalian target of rapamycin (mTOR) [152]	
Nuclear-factor-E2-related factor (Nrf2) [153]	
Ribosomal protein S6 kinase beta-1 (p70S6K) [110]	
Serine/threonine-protein kinase PAK 4 (PAK4) [154]	
Protein disulfide isomerase family A, member 3 pseudogene (PDIA3P) [155]	
Phosphatidylinositol-3-kinase (PI-3K) [134]	
Phospholipase C [110]	
Phospholipase C-γ [156]	
Platelet-derived growth factor (PDGF) [156]	
Polo-like kinase 1 (PLK-1) [157]	
Ras-GTPase [68]	
S6 kinase [158]	
Snail [159]	
Sterol-responsive element bindingprotein (SREBP) 1 [68]	
Stobadine [160]	
TAp73 [161]	
Testosterone [110]	
Transforming growth factor beta 1 (TGF-β1) [162]	
TP53-induced glycolysis and apoptosis regulator (TIGAR) [163]	
Vascular endothelial cell growth factor (VEGF) [25]	
Vitamin D [164]	
Vitamin E [160]	

## Figures and Tables

**Table 1:** Classification of G6PD mutations.

Class	Mutation Severity	% of Normal G6PD Function
Class I	Severe deficiency associated with chronic non-spherocytic hemolytic anemia	<1
Class II	Residual activity associated with acute hemolytic anemia	1–10
Class III	Mild	10–60
Class IV	Normal activity	60–150
Class V	More than normal activity	>150
